# Strain-Tunable Gas Sensing Properties of Ag- and Au-Doped SnSe_2_ Monolayers for the Detection of NO, NO_2_, SO_2_, H_2_S and HCN

**DOI:** 10.3390/nano15181454

**Published:** 2025-09-21

**Authors:** Yulin Ma, Danyi Zhang, Zhao Ding, Kui Ma

**Affiliations:** 1 Department of Electronic Science, Guizhou University, Guiyang 550025, China; mayulin@zuaa.zju.edu.cn (Y.M.);; 2 Key Laboratory of Micro-Nano-Electronics and Software Technology of Guizhou Province, Guiyang 550025, China; 3 Semiconductor Power Device Reliability Engineering Research Center of Ministry of Education, Guiyang 550025, China

**Keywords:** SnSe_2_ monolayer, noble metal doping, gas sensing, strain engineering, first-principles calculations

## Abstract

In this work, the gas sensing properties and adsorption mechanisms of Ag- and Au-doped SnSe_2_ monolayers toward NO, NO_2_, SO_2_, H_2_S, and HCN were systematically investigated via first-principles calculations. The results demonstrate that NO_2_ exhibits the strongest interaction and the highest charge transfer in both doped systems, indicating superior sensing selectivity. Biaxial strain (ranging from −8% to 6%) was further applied to modulate adsorption behavior. By evaluating changes in equilibrium height, adsorption energy, charge transfer, and recovery time across ten representative adsorption systems, it was found that both compressive and tensile strains enhance the interaction between gas molecules and doped SnSe_2_ monolayers. Specifically, H_2_S/Au–SnSe_2_ and HCN/Au–SnSe_2_ are highly sensitive to tensile strain, while NO/Au–SnSe_2_, H_2_S/Ag–SnSe_2_, NO/Ag–SnSe_2_, and NO_2_/Ag–SnSe_2_ respond more strongly to compressive strain. Systems such as NO_2_/Au–SnSe_2_, SO_2_/Au–SnSe_2_, and SO_2_/Ag–SnSe_2_ respond to both types of strain, whereas HCN/Ag–SnSe_2_ shows relatively low sensitivity in charge transfer. Recovery time analysis indicates that NO_2_ exhibits the slowest desorption kinetics and is most affected by strain modulation. Nevertheless, increasing the operating temperature or applying appropriate strain can significantly shorten recovery times. While other gas systems show smaller variations, strain engineering remains an effective strategy to tune desorption behavior and enhance overall sensor performance. These findings offer valuable insights into strain-tunable gas sensing behavior and provide theoretical guidance for the design of high-performance gas sensors based on two-dimensional SnSe_2_ materials.

## 1. Introduction

Harmful gases such as nitrogen oxides (NO_x_), sulfur dioxide (SO_2_), hydrogen cyanide (HCN), and hydrogen sulfide (H_2_S) pose serious threats to both human health and the environment [1]. Among them, SO_2_ and NO_x_ are primarily released from fossil fuel combustion in power plants and industrial processes, contributing to acid rain and ecological degradation. Toxic gases like HCN and H_2_S are known for their flammable, corrosive, and irritating nature, leading to severe health hazards even at low concentrations. Therefore, the development of effective and sensitive gas sensors, particularly those based on two-dimensional (2D) nanomaterials, has become a crucial research focus for real-time monitoring and early warning of hazardous gases.

2D layered nanomaterials have attracted significant attention for gas sensing applications due to their high surface-to-volume ratios, abundant reactive edge sites, and easily tunable electronic properties [2,3,4]. Their sensing performance can be effectively modulated through surface engineering approaches such as doping or defect introduction. Among various candidates, tin diselenide (SnSe_2_), a typical member of the metal dichalcogenide (MDC) family, features a layered structure, strong in-plane anisotropy, and high carrier mobility. In the realm of gas sensing, SnSe_2_-based materials have shown great potential. Other studies have demonstrated enhanced gas sensing performance through elemental doping and vacancy engineering. Notably, numerous first-principles calculation studies have demonstrated that its sensing capabilities can be further enhanced via elemental doping and vacancy engineering, as summarized in Table 1.

In experiments, Cao et al. synthesized SnSe_2_ and its derived heterojunctions (e.g., SnO_2_/SnSe_2_, Au-SnO_2_/SnSe_2_) via solvothermal and thermal treatments for NO_2_ sensing [10]. Experiments showed that the SnO_2_/SnSe_2_ heterojunction exhibited a response of 15.07 with response/recovery times of 63 s/122 s and a detection limit of 60.61 ppb at 90 °C under 4 ppm NO_2_. The Au-SnO_2_/SnSe_2_ heterojunction showed an even higher response of 25.31, response/recovery times of 156 s/56 s, and a detection limit of 13.67 ppb at 80 °C. The enhanced performance is attributed to improved interfacial electron transfer, increased surface area, and the spill-over effect. Han et al. demonstrated a low-temperature formaldehyde sensor based on a hydrothermally synthesized SnO_2_/SnSe_2_ nanostructure [11]. SnO_2_ nanosheets were decorated on SnSe_2_ nanoblocks, and microstructural characterization confirmed this unique morphology. At 150 °C, the SnO_2_/SnSe_2_ sensor exhibited a response of 13.47 to 10 ppm formaldehyde, with response/recovery times of 63 s/12 s, more than three times higher than that of pure SnO_2_. The sensor also enabled ppb-level detection, showing excellent repeatability, long-term stability, and humidity resistance. The enhanced performance is attributed to the n–n heterojunction between SnO_2_ and SnSe_2_ and its unique microstructure. Rani et al. employed the thermal evaporation method to fabricate an n-SnSe_2_/p-SnO/n-SnSe heterojunction for NO_2_ sensing at room temperature (RT) [12]. At RT, the sensor exhibited a response of 256% to 5 ppm NO_2_, with response/recovery times of 34 s/272 s, and a calculated detection limit of ~115 ppb (38% response).

Beyond compositional tuning, external stimuli such as strain and electric fields also provide effective strategies to tailor gas sensing properties. Zhao et al. reported that strain and electric fields significantly affect the adsorption energy and charge transfer in NO_2_-adsorbed monolayer and bilayer SnS_2_ systems [13]. Although Ag-doped SnSe_2_ films have been synthesized and their sensing capabilities toward CO and NO gases have been experimentally verified [14], a comprehensive theoretical investigation on the gas sensing behavior of noble metal (Ag, Au) doped SnSe_2_ monolayers, particularly under strain, remains limited.

While these studies provide valuable insights, a systematic understanding of noble-metal (Ag, Au) doped SnSe_2_ monolayers toward multiple hazardous gases under both unstrained and strained conditions remains limited. In particular, electronic interactions, adsorption/desorption kinetics, and recovery characteristics have not been comprehensively analyzed. In this study, we address these gaps by performing first-principles calculations of Ag- and Au-doped SnSe_2_ monolayers interacting with NO, NO_2_, SO_2_, H_2_S, and HCN, evaluating adsorption energies, charge transfer, and electronic interactions via charge density difference (CDD) and density of states (DOS) analyses. We further explore the effect of biaxial strain on adsorption and recovery behavior, allowing prediction of sensor performance and providing mechanistic insights for designing high-performance, strain-tunable 2D gas sensors. Key novelties of this work include: (1) systematic comparison of Ag- and Au-doped SnSe_2_ monolayers for multiple hazardous gases; (2) comprehensive analysis of electronic interactions, adsorption energies, charge transfer, and recovery times; (3) investigation of biaxial strain effects on adsorption/desorption behavior to guide strain-engineered sensor design; and (4) bridging first-principles calculations with practical sensor performance trends. Overall, this work provides a comprehensive framework for understanding and designing high-performance 2D gas sensors, addressing limitations of previous studies that considered only single gases or limited modifications.

## 2. Calculation Model and Methods

**Figure 1:**
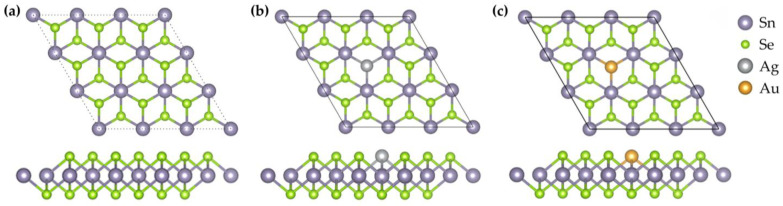
Top and side views of the optimized SnSe_2_ (3 × 3 × 1) supercells: (**a**) pristine; (**b**) Ag-doped with Ag substituting a Se atom; (**c**) Au-doped with Au substituting a Se atom.

All first-principles calculations in this study were performed using the CASTEP [15]. The exchange–correlation interactions were treated within the generalized gradient approximation (GGA) using the Perdew–Burke–Ernzerhof (PBE) functional [16]. In addition, to compensate for the inherent limitations of GGA in accurately describing weak interactions such as van der Waals forces, the van der Waals interactions between the Ag- and Au-doped SnSe_2_ monolayer and gas molecules were considered and corrected using the dispersion-corrected DFT-D3 method [17]. To construct the doping model, a 3 × 3 × 1 supercell of monolayer SnSe_2_ was employed, in which a single Se atom was substituted with a noble metal atom (Ag or Au), corresponding to a nominal doping concentration of ~3.7%. Although this concentration is higher than typical experimentally achievable dilute levels, it is primarily dictated by the finite supercell size and represents a practical compromise between physical reliability and computational feasibility in terms of CPU time and memory requirements. Nevertheless, high-concentration theoretical calculations are valuable because they amplify the effects of doping and clarify the underlying physical and chemical trends, such as the influence of dopants on the band structure, Fermi-level position, and gas adsorption behavior, which generally persist even at lower concentrations. At experimentally accessible dilute levels, dopant-induced electronic states are expected to become more localized, band-gap modifications weaker, and molecule–surface interactions reduced, leading to lower adsorption energies and charge transfer, although selectivity may in some cases improve. During structural optimization, the energy cutoff was set at 500 eV, and the k-point sampling grid was configured as 5 × 5 × 1. To eliminate interlayer interactions along the z-direction due to periodic boundary conditions, a vacuum layer of 20 Å was introduced. The final optimized monolayer structure contained 27 atoms, including 9 Sn atoms and 18 Se atoms, as illustrated in Figure 1.

For gas adsorption studies, the initial distance between the gas molecule and the surface of the Ag- or Au-doped SnSe_2_ monolayer was set to 3 Å. The adsorption energy ($E_{ad}$) was calculated to evaluate the thermodynamic stability of gas adsorption, and is defined as:

(1) $$E_{ad}=E_{monolayer-gas}-E_{monolayer}-E_{gas}$$

where $E_{monolayer-gas}$, $E_{monolayer}$, and $E_{gas}$ represent the total energies of the adsorption system, the isolated Ag (or Au)-doped SnSe_2_ monolayer, and the isolated gas molecule, respectively. A negative $E_{ad}$ value indicates an exothermic and energetically favorable adsorption process.

To further investigate the electronic interactions between gas molecules and the substrate, CDD analysis was carried out. The differential charge density (Δρ) is expressed as:

(2) $$\Delta \rho =\rho_{monolayer-gas}-\rho_{monolayer}-\rho_{gas}$$

where $\rho_{monolayer-gas}$, $\rho_{monolayer}$, and $\rho_{gas}$ represent the charge densities of the adsorption system, the pristine Ag (or Au)-doped SnSe_2_ monolayer, and the isolated gas molecule, respectively. Notably, $\rho_{monolayer}$ and $\rho_{gas}$ were computed using the same atomic configuration as in the adsorption complex to ensure accuracy. Charge transfer analysis serves as a critical indicator of the interaction strength and sensing behavior of gas molecules on 2D materials [18].

## 3. Results

### 3.1. Ag- and Au-Doped SnSe_2_ Monolayers

**Figure 2:**
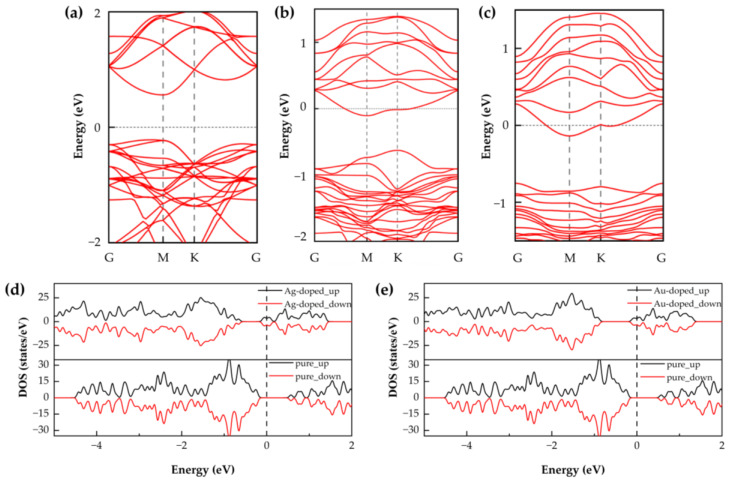
Electronic properties of pristine and noble metal-doped SnSe_2_ monolayers: (**a**) Band structure of pristine SnSe_2_; (**b**) Band structure of Ag-doped SnSe_2_; (**c**) Band structure of Au-doped SnSe_2_; (**d**) Total DOS (TDOS) of pristine and Ag-doped SnSe_2_; (**e**) TDOS of pristine and Au-doped SnSe_2_.

As illustrated in Figure 2, the SnSe_2_ monolayers doped with noble metal atoms (Ag or Au) were fully optimized, and their structural and electronic properties were analyzed. To investigate the gas-sensing characteristics, we calculated the band structures and DOS of pristine SnSe_2_ as well as those of Ag- and Au-doped systems. The calculated band gap of pristine SnSe_2_ was 0.785 eV. The introduction of Ag or Au atoms into the SnSe_2_ lattice significantly alters its electronic structure. In both doped systems, the conduction band crosses the Fermi level, indicating a transition from semiconducting to semimetallic behavior. This change implies enhanced conductivity, which may contribute to improved gas-sensing performance [8].

### 3.2. Gas Adsorption Behavior

**Figure 3:**
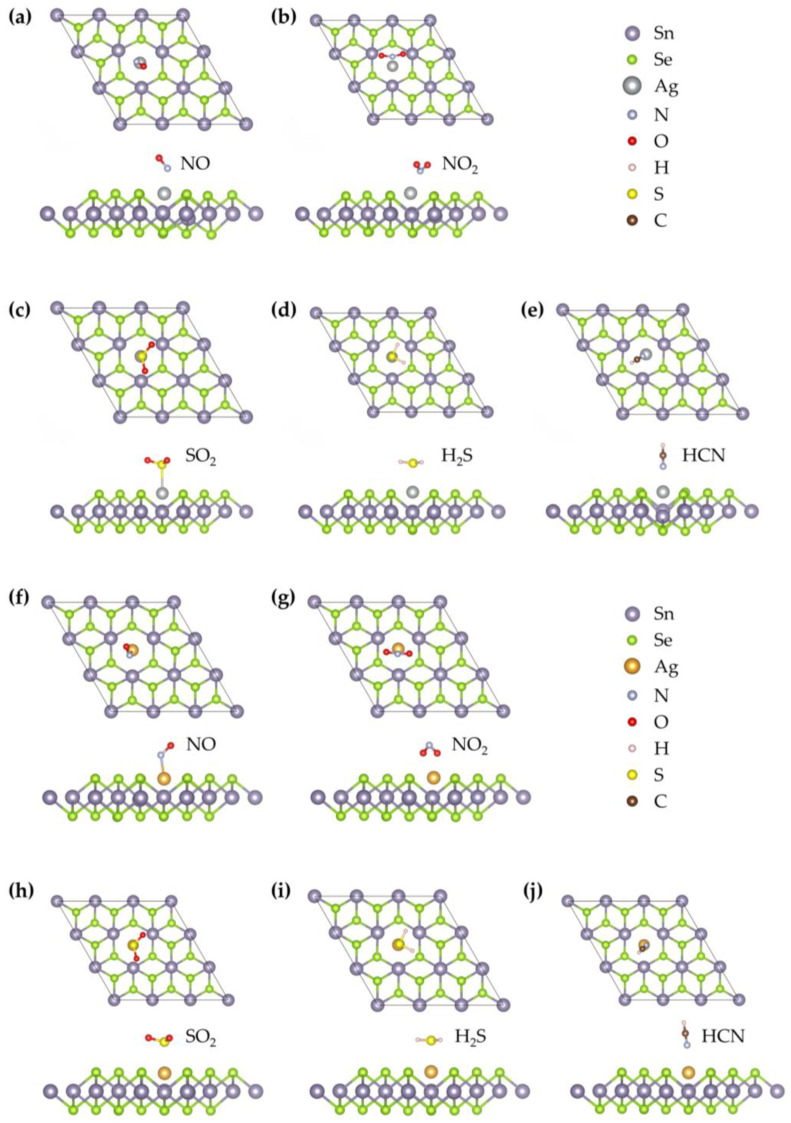
Optimized top and side views of five gas molecules adsorbed on (**a**–**e**) Ag-doped and (**f**–**j**) Au-doped SnSe_2_ monolayers: (**a**,**f**) NO; (**b**,**g**) NO_2_; (**c**,**h**) SO_2_; (**d**,**i**) H_2_S; (**e**,**j**) HCN.

We evaluated the adsorption configurations of five hazardous gases (NO, NO_2_, SO_2_, H_2_S, and HCN) on noble metal-doped SnSe_2_ monolayers, focusing on adsorption sites near the Ag or Au atoms. The most stable configurations for each gas molecule are depicted in Figure 3, corresponding to the Ag- and Au-doped systems, respectively. Upon doping, the optimized bond lengths of the adsorbed gas molecules (NO, NO_2_, SO_2_, H_2_S, and HCN) varied depending on the dopant. For the Ag–SnSe_2_ system, the bond lengths were 1.176 Å (NO), 1.237 Å (NO_2_), 1.453 Å (SO_2_), 1.157 Å (H_2_S), and 1.075 Å (HCN). For the Au–SnSe_2_ system, they were 1.179 Å (NO), 1.264 Å (NO_2_), 1.454 Å (SO_2_), 1.352 Å (H_2_S), and 1.075 Å (HCN), respectively. These changes suggest varying degrees of interaction between the gas molecules and the doped surfaces.

The key adsorption parameters for the five gases on Ag–SnSe_2_ and Au–SnSe_2_ are summarized in Table 2. To better understand the sensing behavior, we computed the $E_{ad}$, equilibrium height (the vertical distance between the gas molecule and the monolayer), and charge transfer between the gas molecules and the substrate. $E_{ad}$ is a critical parameter that reflects the interaction strength between the gas molecule and the surface. A negative adsorption energy indicates a thermodynamically favorable process. In our results, all five gases (NO, NO_2_, SO_2_, H_2_S, and HCN) exhibited negative adsorption energies on both Ag- and Au-doped SnSe_2_ surfaces, confirming spontaneous and stable adsorption. Moreover, the absolute values of the adsorption energy for most gas molecules exceeded 0.30 eV, which suggests strong adsorption and high sensing potential. Among them, NO_2_ showed the most pronounced interaction with both doped substrates, indicating its potential as a highly detectable target gas. These findings provide a theoretical foundation for further analyzing the gas adsorption mechanisms of NO, NO_2_, SO_2_, H_2_S, and HCN on Ag–SnSe_2_ and Au–SnSe_2_ monolayers.

To distinguish physisorption from chemisorption in this study, adsorption energy, charge transfer, equilibrium distance, and electronic structure were jointly considered. Adsorption energy serves as a primary quantitative indicator: weakly negative values (${|E}_{ad}|$ ≤ 0.5 eV) typically correspond to physisorption dominated by van der Waals forces, while larger negative values (${|E}_{ad}|$ > 0.5 eV) suggest chemisorption with stronger covalent or ionic interactions. Charge transfer between the adsorbate and substrate was quantified using Bader or Mulliken analysis, where minimal transfer (ΔQ ≤ 0.05 e) indicates physisorption and significant transfer (ΔQ > 0.1 e) correlates with chemisorption. The equilibrium distance between the molecule and substrate also provides insight, with larger distances (>2.5 Å) indicating physisorption and shorter distances (<2.2–2.3 Å) suggesting chemical bonding. Finally, total and projected density of states (DOS/PDOS) analyses were used to evaluate orbital interactions; physisorption usually induces minor orbital shifts and weak overlap without new states near the Fermi level, whereas chemisorption is often accompanied by strong orbital hybridization, formation of new states, and significant electronic structure changes. By combining these criteria, NO and NO_2_ adsorption on noble metal-doped SnSe_2_ is generally classified as chemisorption due to their larger adsorption energies and moderate charge transfer, while H_2_S and HCN adsorption exhibits low adsorption energies, minimal charge transfer, and weak orbital overlap, indicating physisorption.

#### 3.2.1. NO Adsorption

**Figure 4:**
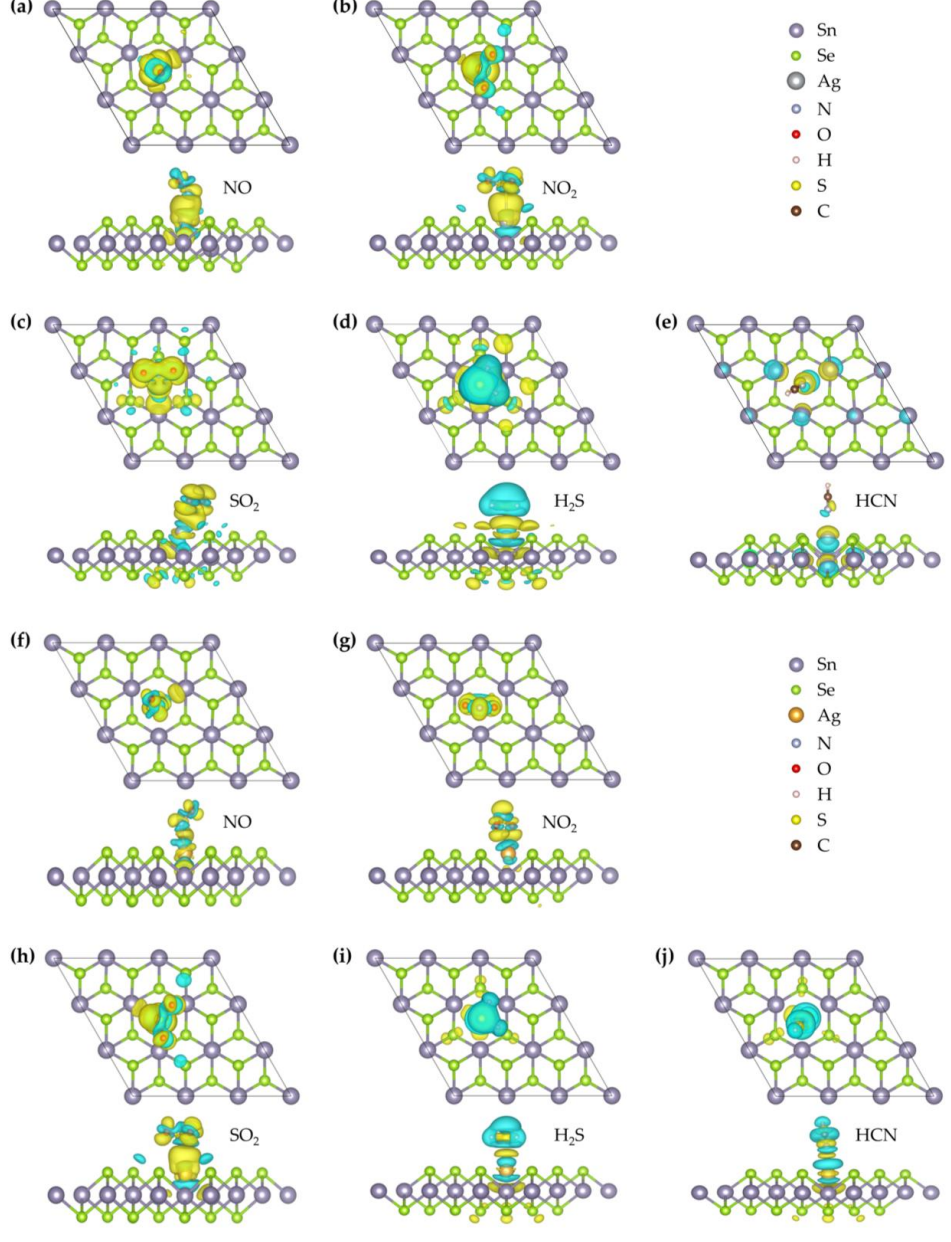
Top and side views of CDD plots for five gas molecules adsorbed on (**a**–**e**) Ag-doped and (**f**–**j**) Au-doped SnSe_2_ monolayers: (**a**,**f**) NO; (**b**,**g**) NO_2_; (**c**,**h**) SO_2_; (**d**,**i**) H_2_S; (**e**,**j**) HCN.

**Figure 5:**
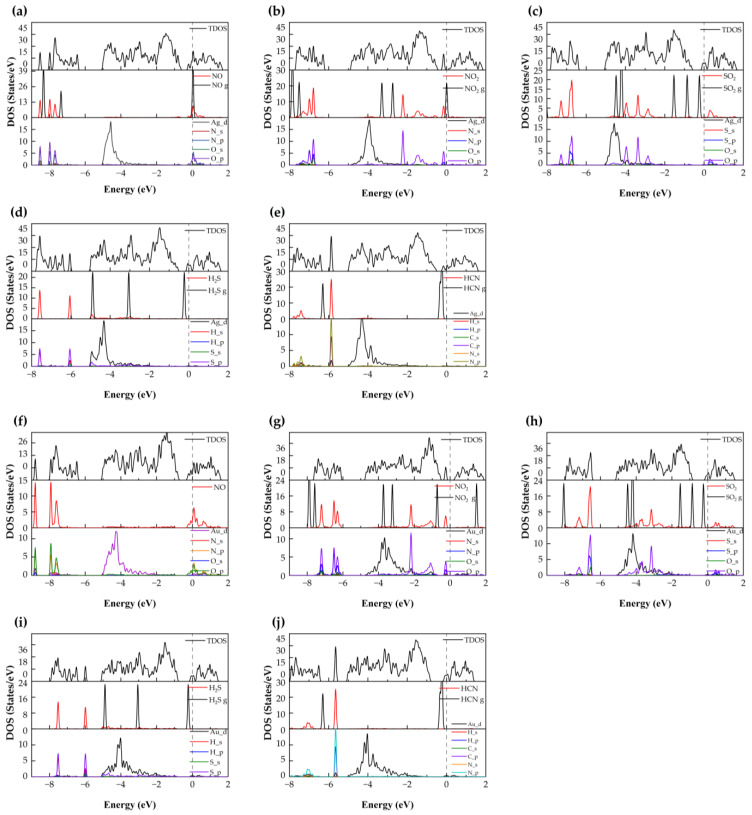
TDOS and PDOS plots of five gas molecules adsorbed on (**a**–**e**) Ag-doped and (**f**–**j**) Au-doped SnSe_2_ monolayers: (**a**,**f**) NO; (**b**,**g**) NO_2_; (**c**,**h**) SO_2_; (**d**,**i**) H_2_S; (**e**,**j**) HCN.

As shown in Figure 3, the optimized stable configuration of NO on the Ag–SnSe_2_ monolayer corresponds to a tilted, nearly parallel orientation above the Ag atom. The N–O bond length of a free NO molecule is 1.15 Å, and adsorption slightly elongates this bond by about 0.026 Å, indicating a minor weakening of the N–O bond. The equilibrium distance between the NO molecule and the substrate is 2.104 Å, and the adsorption energy is −0.29 eV. According to commonly adopted criteria, adsorption energies below about −0.5 eV, charge transfers less than 0.2 e, and molecule–surface distances larger than 2 Å are generally indicative of physisorption. Consistently, Bader charge analysis shows that only ~0.14 e is transferred from NO to the Ag–SnSe_2_ surface, supporting a physisorption-dominated interaction. To further examine the electronic features, the CDD and DOS were analyzed. As illustrated in Figure 4, charge accumulation is observed between the N atom of NO and the Ag atom, and slight orbital overlaps between Ag-4d, N-2p, and O-2p states occur at around −8.53 eV, −7.97 eV, and −7.68 eV (Figure 5). However, no distinct new states emerge near the Fermi level, and the metallic nature of the doped system remains unchanged. These results suggest that although limited orbital hybridization exists, the overall interaction between NO and the Ag–SnSe_2_ monolayer is weak and primarily governed by physisorption, which may still induce detectable but moderate modulation of the electronic properties relevant for sensing applications.

As shown in Figure 3, the most stable configuration of NO adsorption on the Au–SnSe_2_ monolayer corresponds to a tilted orientation of the molecule relative to the surface. The bond length of a free NO molecule is 1.151 Å, which slightly elongates by about 0.028 Å upon adsorption, suggesting a minor weakening of the N–O bond. The equilibrium distance between the NO molecule and the substrate is 2.092 Å, and the calculated adsorption energy is −0.37 eV. Although this value is larger in magnitude than that for NO adsorption on Ag–SnSe_2_, it still lies near the boundary between weak chemisorption and strong physisorption. To further probe the interaction mechanism, we analyzed the CDD, Bader charge transfer, and DOS. As shown in Figure 4, charge accumulation is observed around the NO molecule, while charge depletion occurs around the neighboring Au atom, indicating electron redistribution between the adsorbate and substrate. Bader analysis reveals that approximately 0.171 e is transferred from the substrate to the NO molecule, implying a modest degree of charge transfer. The TDOS and projected density of states (PDOS) results (Figure 5) confirm that the metallic nature of the doped system is preserved after adsorption. The molecular orbitals of NO shift slightly toward lower energies, with overlapping features between Au-5d, O-2p, and N-2p states around −8.8 eV and −7.93 eV. These hybridization signatures, together with the moderate adsorption energy and limited charge transfer, suggest that the interaction involves weak chemisorption features, while still retaining characteristics of physisorption. Overall, NO adsorption on Au–SnSe_2_ exhibits an intermediate binding nature, which may influence its sensing performance by providing detectable electronic perturbations without strongly immobilizing the molecule.

#### 3.2.2. NO_2_ Adsorption

As summarized in Table 2, the adsorption energy of NO_2_ on the Ag–SnSe_2_ monolayer is calculated to be −1.03 eV, indicating a strong interaction that is characteristic of chemisorption. This result contrasts with previous reports on pristine SnSe_2_, where Cheng et al. obtained an adsorption energy of only −0.29 eV for NO_2_, corresponding to physisorption [9]. The most stable configuration of NO_2_ on Ag–SnSe_2_ adopts a V-shaped geometry with both O atoms pointing upward, located at an equilibrium distance of 1.96 Å from the substrate. For a free NO_2_ molecule, the N–O bond lengths are 1.21 Å and 1.209 Å, which increase slightly by about 0.02–0.03 Å upon adsorption, reflecting a weakening of the original N–O bonds. The charge density difference (Figure 4) reveals a large accumulation of electrons around the NO_2_ molecule, further corroborated by Bader charge analysis, which shows that approximately 0.54 e is transferred from the substrate to NO_2_. Figure 5 presents the TDOS, a comparison between the DOS of free and adsorbed NO_2_, and the PDOS of the interacting system. The molecular orbitals of NO_2_ shift toward lower energies upon adsorption, with distinct peaks appearing near the Fermi level. Combined with the band structure, these features indicate that the doped system undergoes a transition from metallic to semiconducting behavior. Moreover, hybridization peaks between Ag-4d and O-2p states confirm orbital interactions, supporting the conclusion that NO_2_ adsorption on Ag–SnSe_2_ involves strong chemisorption.

For NO_2_ adsorption on the Au–SnSe_2_ monolayer, structural optimization starting from a parallel orientation yields a stable configuration in which the NO_2_ molecule is nearly perpendicular to the substrate, with the two O atoms located below the N atom. The N–O bond lengths elongate to 1.264 Å, and the equilibrium distance between the molecule and the surface is 2.10 Å. The differential charge density (Figure 4) reveals significant electron accumulation around the NO_2_ molecule accompanied by charge depletion around the Au atom, consistent with strong charge transfer. Bader analysis quantifies a transfer of approximately 0.654 e from the substrate to NO_2_. The adsorption energy is calculated to be −1.12 eV, the largest among all studied gases, indicating highly stable adsorption. In the DOS and PDOS results (Figure 5), the system exhibits a transition from metallic to semiconducting character after adsorption. The molecular orbitals of NO_2_ shift toward lower energies, with pronounced hybridization peaks between Au-5d, N-2p, and O-2p states observed at −7.21 eV, −6.49 eV, −2.19 eV, and −0.24 eV. Compared with NO adsorption, the larger charge transfer and stronger orbital hybridization further confirm that NO_2_ adsorption on Au–SnSe_2_ involves chemisorption. According to the combined criteria of adsorption energy (${|E}_{ad}|$ > 0.5 eV), charge transfer (>0.2 e), short equilibrium distance (<2.5 Å), and clear DOS hybridization, this adsorption can be reliably categorized as chemisorption.

#### 3.2.3. SO_2_ Adsorption

As shown in Figure 3, the optimized adsorption configuration of SO_2_ on the Ag–SnSe_2_ monolayer features the molecule aligned nearly parallel to the substrate, positioned above the Ag atom, with an equilibrium distance of 2.65 Å. The charge density difference indicates minor electron accumulation around the SO_2_ molecule and between the molecule and the Ag atom. Bader charge analysis reveals that the SO_2_ molecule gains only about 0.05 e from the substrate, consistent with weak charge transfer. Together with the adsorption energy (Table 2), these results suggest that the adsorption is dominated by physisorption. The DOS and PDOS analysis further supports this conclusion: the molecular orbitals of SO_2_ broaden and weaken after adsorption, mainly within −7.88 to −6.35 eV and −4.51 to −2.03 eV, while additional weak resonances appear near the conduction band edge (0.21–0.88 eV). Compared with the free SO_2_ molecule, the absorption peaks near the Fermi level are diminished after adsorption. A weak hybridization feature between Ag-4d and O-2p states is observed around −4.88 to −3.83 eV, indicating limited orbital overlap. Overall, based on adsorption energy (<−0.5 eV), minimal charge transfer, and a relatively large equilibrium distance, the SO_2_ adsorption on Ag–SnSe_2_ can be reliably classified as physisorption, although the slight orbital hybridization may still influence sensing response.

After structural relaxation, the SO_2_ molecule adopts a tilted V-shaped configuration above the Au–SnSe_2_ monolayer with an equilibrium distance of 2.657 Å. The S–O bond lengths increase slightly from 1.43 Å to 1.454 Å, suggesting a small weakening of the intramolecular bonds. As shown in Figure 4, the charge density difference indicates electron accumulation around the SO_2_ molecule and the substrate surface. Bader charge analysis reveals that approximately 0.225 e is transferred from the substrate to the molecule, indicating limited charge transfer. The adsorption energy of −0.15 eV further supports that the interaction is dominated by physisorption. To better understand the electronic influence of adsorption, the TDOS and PDOS were analyzed (Figure 5). Compared with the free molecule, the orbitals of adsorbed SO_2_ shift toward lower energies, and the sharp peaks are slightly reduced, indicating orbital broadening. Weak hybridization features involving Au-d, S-p, and O-p states are observed near −7.72 eV, −6.56 eV, and −5.20 to −0.89 eV. Overall, the low adsorption energy, small charge transfer, and relatively large adsorption distance consistently indicate that SO_2_ adsorption on Au–SnSe_2_ is physisorption with only minor orbital interactions.

#### 3.2.4. H_2_S Adsorption

After structural optimization, the H_2_S molecule stably adsorbs above the Ag atom on the Ag–SnSe_2_ monolayer at an equilibrium distance of 2.61 Å, with its molecular plane nearly parallel to the substrate. The H–S bond lengths slightly shorten to 1.352 Å and 1.353 Å, indicating minimal intramolecular changes. The adsorption energy is calculated to be −0.57 eV, suggesting a stronger interaction than that of NO, but still indicative of weak chemisorption or strong physisorption. The charge density difference (Figure 4) shows electron depletion around the H_2_S molecule and accumulation in the Ag–SnSe_2_ substrate. Bader analysis quantifies a charge transfer of approximately 0.16 e from the H_2_S molecule to the substrate. TDOS and PDOS analysis (Figure 5) indicates that the molecular orbitals of H_2_S shift slightly toward lower energies upon adsorption, with peaks appearing at −7.85 to −7.45 eV and −6.25 to −5.86 eV, contributing modestly to the total density of states. Weak orbital hybridization between Ag-d and S-p states is observed, consistent with limited electronic interaction. Overall, based on adsorption energy, charge transfer, and equilibrium distance, H_2_S adsorption on Ag–SnSe_2_ can be categorized as weak chemisorption or strong physisorption.

After structural optimization, the H_2_S molecule adopts a parallel configuration above the Au atom on the Au–SnSe_2_ monolayer, with an equilibrium distance of 2.66 Å. The S–H bond lengths remain nearly unchanged at 1.352 Å. The adsorption energy is calculated to be −0.41 eV, indicating a weak interaction between H_2_S and the substrate. Charge density difference analysis (Figure 5) shows electron depletion around the H_2_S molecule, with a Bader charge transfer of only 0.041 e from the molecule to the substrate. TDOS analysis indicates negligible changes near the Fermi level after adsorption. Comparing the DOS of free and adsorbed H_2_S molecules, the molecular orbitals shift slightly to lower energies, and peak intensities are reduced. Weak overlap between H-p and S-p orbitals is observed at −7.52 eV and −5.99 eV, suggesting minimal orbital hybridization. Taken together, the low adsorption energy, minimal charge transfer, and minor orbital interactions indicate that H_2_S adsorption on Au–SnSe_2_ is physisorption.

#### 3.2.5. HCN Adsorption

After optimization, the HCN molecule adsorbs on the Ag–SnSe_2_ monolayer with the molecule tilted toward the nearby Se atom and positioned above the Ag atom at an equilibrium distance of 2.24 Å, as shown in Figure 3. The adsorption energy is −0.508 eV, and Bader analysis indicates an extremely small charge transfer of 0.004 e from the HCN molecule to the substrate. The charge density difference shows minimal electron redistribution between HCN and the Ag–SnSe_2_ monolayer, confirming that the interaction is very weak. TDOS and PDOS analysis (Figure 5) reveals that the molecular orbitals of adsorbed HCN shift slightly toward lower energies compared to the free molecule, with minor overlaps observed between N-p, C-p, and Ag-d orbitals at −5.87 eV and between H-s, C-p, and N-p orbitals in the −7.94 to −7.31 eV range. Overall, the low adsorption energy, negligible charge transfer, and minimal orbital overlap indicate that HCN adsorption on Ag–SnSe_2_ is dominated by physisorption.

After structural optimization, the HCN molecule adsorbs slightly tilted above the Au atom on the Au–SnSe_2_ monolayer, with the N atom closest to the substrate at a distance of 2.405 Å. The C–N and C–H bond lengths are 1.159 Å and 1.076 Å, respectively, showing minor elongation compared with the free molecule. The adsorption energy is −0.23 eV, indicating very weak interaction. Charge density analysis (Figure 4) shows minimal electron redistribution around the HCN molecule, and Mulliken analysis indicates a small charge transfer of 0.043 e from the HCN molecule to the substrate. TDOS and PDOS analyses (Figure 5) reveal that the monolayer retains its metallic character after adsorption. The molecular orbitals of HCN shift slightly toward lower energies, and a minor localized peak appears at −5.64 eV, mainly contributed by N-p and H-p orbitals with slight contribution from Au-d orbitals, indicating weak electronic localization. Taken together, the low adsorption energy, minimal charge transfer, and limited orbital interaction confirm that HCN adsorption on Au–SnSe_2_ is physisorption.

### 3.3. Strain Modulation of Gas Adsorption on Ag- and Au-Doped SnSe_2_ Monolayers

#### 3.3.1. Effect of Biaxial Strain on Gas Sensing Performance of Ag-Doped SnSe_2_

To further explore the tunability of adsorption properties, we investigated the influence of external biaxial strain (*ε*) on the gas adsorption behavior of the Ag-doped SnSe_2_ monolayer. The applied strain is defined as:

(3) $$\varepsilon =\frac{\alpha -\alpha_{0}}{\alpha_{0}}$$

where $\alpha_{0}$ and α denote the lattice constants of the unstrained and strained systems, respectively. Positive *ε* corresponds to tensile strain, and negative *ε* indicates compressive strain. A range of biaxial strain from –8% to +6% was applied to evaluate the modulation of equilibrium height, adsorption energy, and charge transfer for each gas molecule.

**Figure 6:**
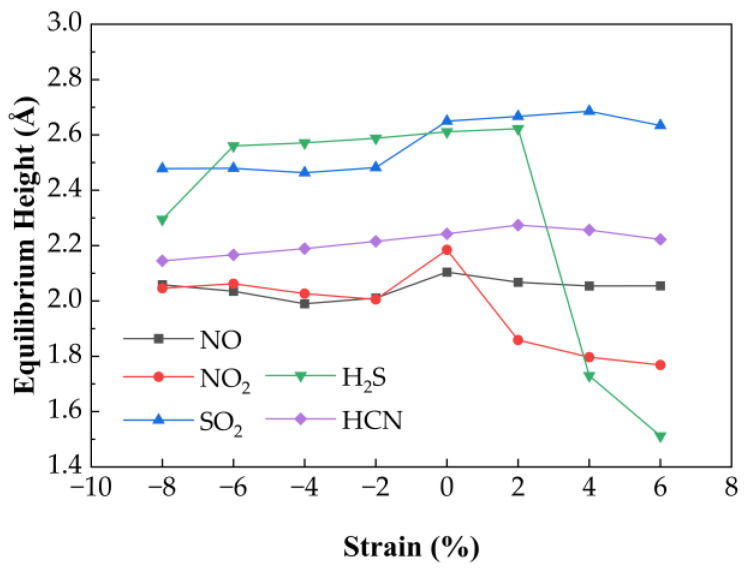
Variation in gas equilibrium heights for five adsorption systems on Ag-doped SnSe_2_ monolayers under biaxial strain ranging from −8% to 6%.

Figure 6 presents the variation in equilibrium heights for NO, NO_2_, SO_2_, H_2_S, and HCN molecules adsorbed on Ag−SnSe_2_ under different strain levels. The equilibrium heights of HCN and NO change only slightly under strain (maximum deviations of 0.03 Å and 0.08 Å, respectively). SO_2_ shows a linear decrease in equilibrium height under compressive strain from −8% to −2%, while remaining relatively stable under tensile strain. In contrast, H_2_S shows a more complex trend: it reaches a minimum height of 2.295 Å at −8%, increases linearly from −6% to +2%, and then drops under higher tensile strain. NO_2_ equilibrium height decreases steadily with both tensile and compressive strain.

**Figure 7:**
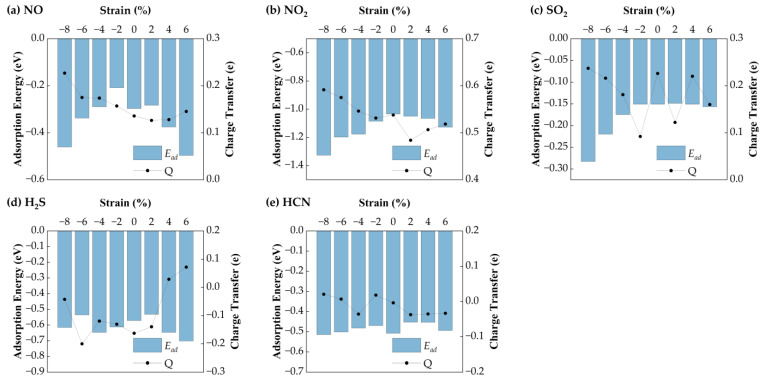
$E_{ad}$ and charge transfer of five gas molecules on the Ag-doped SnSe_2_ monolayer: (**a**) NO; (**b**) NO_2_; (**c**) SO_2_; (**d**) H_2_S; (**e**) HCN.

Figure 7 shows the corresponding adsorption energies under strain. For H_2_S, the adsorption energy remains relatively stable (−0.57 eV), with a maximum variation of 0.11 eV, suggesting weak strain sensitivity. HCN exhibits minimal energy change (~0.05 eV), indicating that strain has little impact on its adsorption stability. In contrast, NO shows a parabolic trend: its adsorption energy increases under compressive strain (−8% to −2%) and decreases with tensile strain, reaching a minimum of −0.30 eV at 6%. NO_2_ displays the strongest strain dependence, with the most favorable adsorption energy (−1.33 eV) at −8%. SO_2_ also shows enhanced adsorption under compressive strain, with a minimum of −0.26 eV at −8%. Bader charge analysis reveals strain-dependent charge transfer behavior. H_2_S initially loses electrons (−0.16 eV) but gains electrons under tensile strain above 2%. HCN shows a reversal: it loses charge under tension and gains under compression. NO exhibits a parabolic charge transfer curve, with the minimum (0.13 eV) at 2% strain. NO_2_ shows a maximum charge transfer of 0.59 eV at −8%. SO_2_ charge transfer decreases linearly under compression but fluctuates under tension.

Overall, H_2_S interacts more strongly with Ag-SnSe_2_ under tensile strain. HCN shows negligible adsorption energy and charge transfer variation, indicating low strain sensitivity. The other gases (NO, NO_2_, SO_2_) exhibit enhanced charge transfer and adsorption energy under compressive strain, suggesting that Ag-doped SnSe_2_ is a promising strain-tunable sensing platform for NO, NO_2_, SO_2_, and to some extent, H_2_S.

#### 3.3.2. Effect of Biaxial Strain on Gas Sensing Performance of Au-Doped SnSe_2_

**Figure 8:**
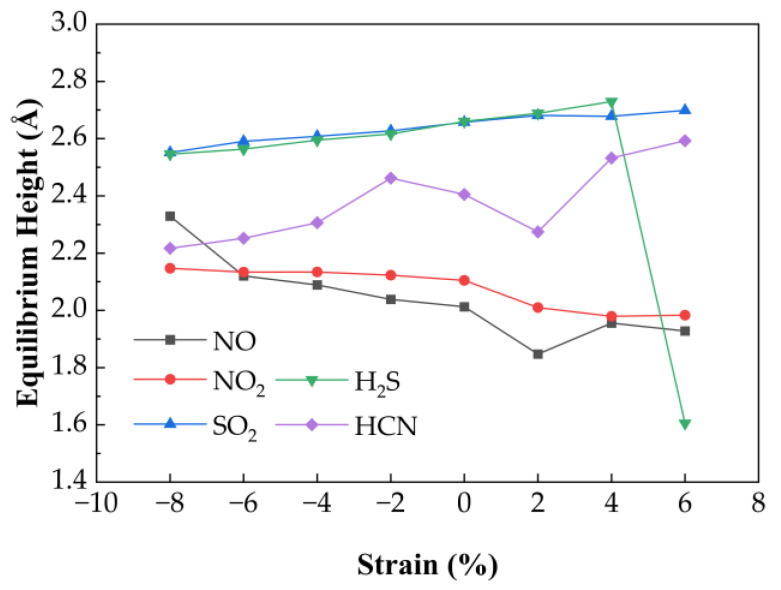
Variation in gas equilibrium heights for five adsorption systems on Au-doped SnSe_2_ monolayers under biaxial strain ranging from −8% to 6%.

The effect of biaxial strain on the gas adsorption properties of Au-doped SnSe_2_ was similarly analyzed. As shown in Figure 8, equilibrium heights of adsorbed gases change slightly under strain. SO_2_, H_2_S, and HCN show moderate increases in height under compression (−8% to 0%) by 0.04 Å, 0.03 Å, and 0.07 Å, respectively. For H_2_S, the height remains ~2.62 Å under moderate strain but drops significantly to 1.61 Å at 6% tensile strain, indicating transition to chemisorption and reduced desorption capability. For NO and NO_2_, equilibrium heights decrease steadily with increasing strain.

**Figure 9:**
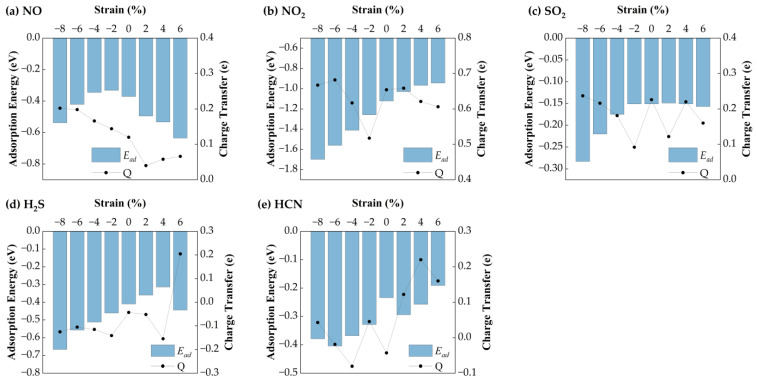
$E_{ad}$ and charge transfer of five gas molecules on the Au-doped SnSe_2_ monolayer: (**a**) NO; (**b**) NO_2_; (**c**) SO_2_; (**d**) H_2_S; (**e**) HCN.

Figure 9 illustrates adsorption energies and charge transfer behavior. H_2_S adsorption energy increases steadily under strain, while HCN shows maximum adsorption at −6%. NO shows a parabolic energy profile with a minimum of −0.635 eV at 6%, indicating strong binding. NO_2_ exhibits the most favorable adsorption at −8% (−0.74 eV), with linear variation across the strain range. SO_2_ also shows enhanced adsorption under compression, with a minimum of −0.284 eV at −8%. All systems exhibit negative adsorption energies, confirming stable physisorption over the full strain range. Charge transfer data further support these findings. H_2_S transitions from electron donor to acceptor beyond 4% strain. HCN undergoes charge reversal across strain states. NO shows a decreasing-to-increasing trend across the strain range, while NO_2_ behaves as a consistent electron acceptor. SO_2_ shows decreasing charge transfer under compression and fluctuating values under tension.

These results suggest that Au-doped SnSe_2_ is a viable candidate for detecting NO, NO_2_, HCN, and SO_2_, with strain-induced modulation enhancing sensitivity, particularly under compressive strain.

### 3.4. Recovery Time

The working principle of semiconductor gas sensors involves electron exchange between the adsorbed gas molecules and the sensing material, resulting in changes in the electrical resistance, which is utilized for gas detection. Therefore, the sensitivity of the sensor is primarily governed by adsorption energy and charge transfer. At a fixed adsorption energy, a greater charge transfer typically enhances the sensor’s response. Moreover, the direction of charge transfer can lead to either an increase or decrease in resistance. 2D layered nanomaterials often exhibit strong interactions with gas molecules, indicating their potential for high sensitivity. However, while strong adsorption is beneficial for detection, excessively strong binding may hinder desorption, adversely affecting the recovery capability of the sensor. Hence, a high-performance gas sensing material must exhibit both sufficient adsorption strength and suitable desorption kinetics, ensuring an appropriate recovery time. Based on transition state theory, the ideal recovery time (τ) can be expressed as:

(4) $$\tau =v_{0}^{-1}exp\left(\frac{E}{kT}\right)$$

where $v_{0}$ is the attempt frequency (typically 10^12^ s^−1^), E is the energy barrier, taken as the absolute value of the adsorption energy $E_{ad}$, k is the Boltzmann constant, and T is the operating temperature. We note that equating the desorption barrier directly to the adsorption energy is a commonly adopted approximation in first-principles studies of 2D gas sensors (e.g., Ref. [8]), and it allows for a qualitative comparison of recovery trends among different gas–substrate systems. Nonetheless, we acknowledge that this approximation may overestimate recovery times because the actual desorption barrier can be affected by kinetic pathways, surface relaxation, or other dynamic effects. Therefore, the calculated recovery times should be interpreted as indicative rather than exact predictions.

**Figure 10:**
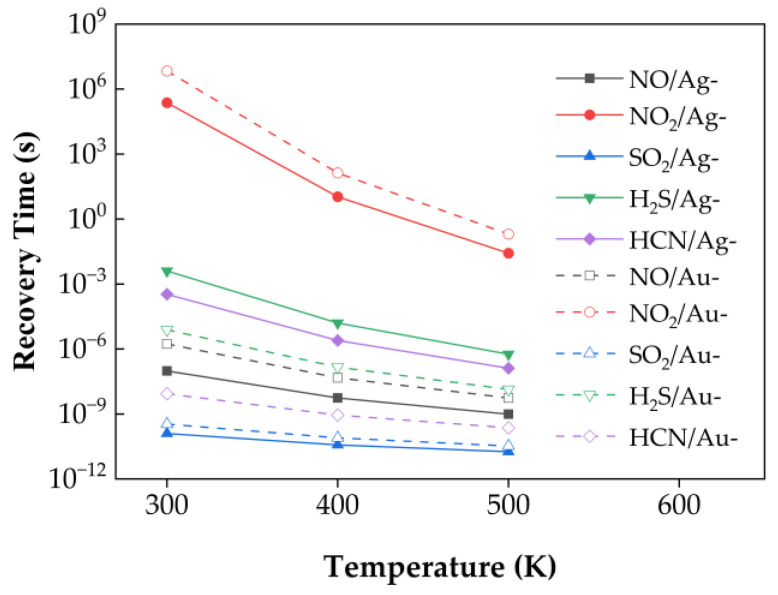
Recovery times of five gas molecules at different temperatures for Ag- and Au-doped SnSe_2_ monolayers.

At room temperature (T = 300 K), kT ≈ 2.585 × 10^−2^ eV. As seen from the equation, recovery time increases exponentially with adsorption energy. Figure 10 presents the calculated recovery times at 300 K, 400 K, and 500 K without applying external strain. At 300 K, the order of recovery time from shortest to longest is: SO_2_/Ag–SnSe_2_, SO_2_/Au–SnSe_2_, HCN/Au–SnSe_2_, NO/Ag–SnSe_2_, NO/Au–SnSe_2_, H_2_S/Au–SnSe_2_, HCN/Ag–SnSe_2_, H_2_S/Ag–SnSe_2_, NO_2_/Ag–SnSe_2_, and NO_2_/Au–SnSe_2_.

At room temperature, some calculated recovery times, particularly for NO_2_, appear relatively long, which may exceed the practical range for typical sensor operation (seconds to minutes). We note that these values are theoretical estimates based on transition state theory and using the adsorption energy as the desorption barrier, and they serve primarily for qualitative comparison of desorption kinetics across different gas–substrate systems. In practice, recovery times can be significantly shortened by elevating the operating temperature or applying external strain, which enhance desorption rates. For instance, increasing the temperature to 400–500 K is expected to reduce NO_2_ recovery times to a feasible range for sensor applications. Therefore, although the room-temperature values seem long, the studied materials remain promising for practical sensing, and the reported trends provide meaningful guidance for designing high-performance 2D gas sensors.

For other gases, such as HCN, SO_2_, NO, and H_2_S, the calculated recovery times at room temperature fall within or near the expected operational range, and the relative trends are consistent with the charge transfer and adsorption energy analysis. Therefore, while the absolute values of the recovery times should be interpreted cautiously, the results provide useful guidance for selecting 2D materials with favorable adsorption and desorption properties for gas sensing.

#### 3.4.1. Recovery Time of NO/Ag–SnSe_2_ and NO/Au–SnSe_2_ Under Strain from −8% to 6% at Different Temperatures

**Figure 11:**
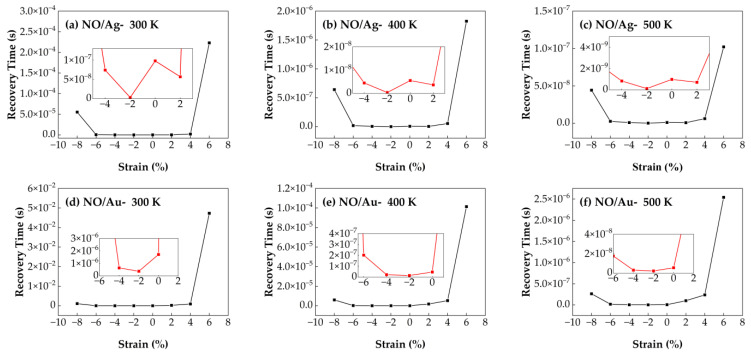
Recovery times of NO adsorption on Ag- and Au-doped SnSe_2_ monolayers under biaxial strains ranging from −8% to 6% at different temperatures: (**a**–**c**) Ag-doped SnSe_2_ at (**a**) 300 K, (**b**) 400 K, and (**c**) 500 K; (**d**–**f**) Au-doped SnSe_2_ at (**d**) 300 K, (**e**) 400 K, and (**f**) 500 K.

Figure 11 shows the recovery time of NO molecules adsorbed on Ag- and Au-doped SnSe_2_ monolayers under biaxial strain ranging from −8% to 6% at different temperatures (300 K, 400 K, and 500 K). The recovery time of NO on Au–SnSe_2_ reaches a minimum of 1.53 s under −2% compressive strain at 400 K, and remains relatively stable across the strain range of −8% to 4%. Similarly, the NO/Ag–SnSe_2_ system also shows its shortest recovery time under −2% strain. However, when the strain exceeds certain thresholds (e.g., −8% or 6%), the recovery time increases significantly, indicating that excessive strain may hinder the desorption process by strengthening gas–substrate interactions. In terms of overall sensing performance, the Ag–SnSe_2_ system demonstrates higher sensitivity toward NO, as evidenced by a shorter recovery time of 1.2 s at −2% strain and 500 K. This suggests that Ag–SnSe_2_ is more suitable for NO gas sensing applications, especially under mild compressive strain and elevated operating temperatures, which facilitate efficient desorption and fast sensor recovery.

#### 3.4.2. Recovery Time of NO_2_/Ag–SnSe_2_ and NO_2_/Au–SnSe_2_ Under Strain from −8% to 6% at Different Temperatures

**Figure 12:**
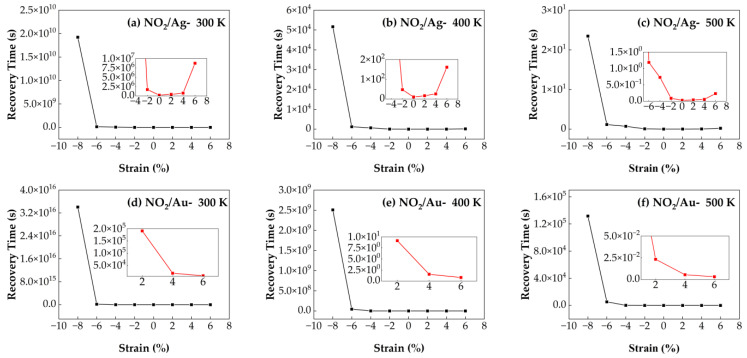
Recovery times of NO_2_ adsorption on Ag- and Au-doped SnSe_2_ monolayers under biaxial strains ranging from −8% to 6% at different temperatures: (**a**–**c**) Ag-doped SnSe_2_ at (**a**) 300 K, (**b**) 400 K, and (**c**) 500 K; (**d**–**f**) Au-doped SnSe_2_ at (**d**) 300 K, (**e**) 400 K, and (**f**) 500 K.

Figure 12 illustrates the recovery times of NO_2_ molecules adsorbed on Ag- and Au-doped SnSe_2_ monolayers under strains ranging from −8% to 6% and at temperatures of 300 K, 400 K, and 500 K. For the NO_2_/Au–SnSe_2_ system, increasing tensile strain from 2% to 6% significantly shortens the recovery time at all examined temperatures, with the shortest recovery time occurring at 6% tensile strain and 500 K. At fixed strain values, the recovery time decreases with increasing temperature, consistent with thermally activated desorption behavior. Under zero strain, the NO_2_/Ag–SnSe_2_ system shows a recovery time of 0.2 s at 500 K, which is shorter than that of the NO_2_/Au–SnSe_2_ system, indicating stronger desorption kinetics. In the NO_2_/Ag–SnSe_2_ system, compressive strain enhances the sensitivity, as demonstrated by the ultra-short recovery time of 0.02 s at 500 K and −2% strain. However, tensile strain reduces the desorption performance of Ag–SnSe_2_ for NO_2_, whereas Au–SnSe_2_ maintains improved recovery times under tensile strain. These findings suggest that compressive strain is detrimental to the overall desorption dynamics in both systems. For Ag–SnSe_2_, optimal NO_2_ sensing performance is achieved under mild compressive strain and elevated temperature. In contrast, tensile strain enhances the recovery behavior in the Au–SnSe_2_ system, indicating different strain modulation strategies are required depending on the choice of dopant.

#### 3.4.3. Recovery Time of SO_2_/Ag–SnSe_2_ and SO_2_/Au–SnSe_2_ Under Strain from −8% to 6% at Different Temperatures

**Figure 13:**
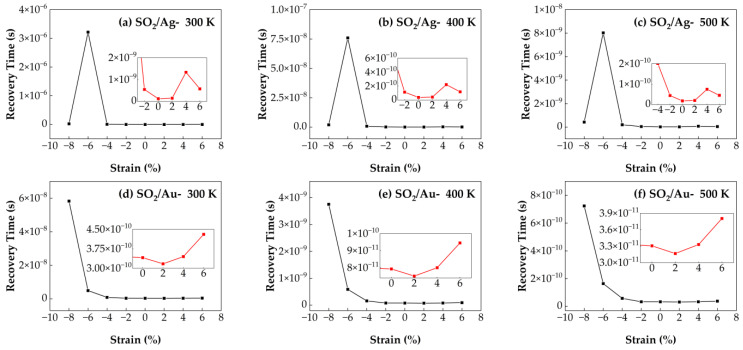
Recovery times of SO_2_ adsorption on Ag- and Au-doped SnSe_2_ monolayers under biaxial strains ranging from −8% to 6% at different temperatures: (**a**–**c**) Ag-doped SnSe_2_ at (**a**) 300 K, (**b**) 400 K, and (**c**) 500 K; (**d**–**f**) Au-doped SnSe_2_ at (**d**) 300 K, (**e**) 400 K, and (**f**) 500 K.

Figure 13 presents the calculated recovery times for SO_2_ molecules adsorbed on Ag- and Au-doped SnSe_2_ monolayers under strain conditions ranging from −8% to 6% and at three temperatures (300 K, 400 K, and 500 K). For the SO_2_/Au–SnSe_2_ system, the maximum recovery time occurs at −8% compressive strain, indicating strong gas–substrate interactions that hinder desorption. In contrast, the shortest recovery time is observed under 2% tensile strain, suggesting that moderate tensile strain can facilitate faster desorption. At fixed strain levels, increasing the temperature significantly reduces recovery time, consistent with the thermal activation mechanism of desorption. Specifically, tensile strain has a relatively minor impact on desorption behavior, whereas compressive strain substantially impedes gas release, especially at lower temperatures. In the SO_2_/Ag–SnSe_2_ system, tensile strain has negligible influence on recovery time. However, under −6% compressive strain, strong adsorption results in an extended recovery time. This effect can be mitigated by elevating the operating temperature. For example, at 500 K without applied strain, the SO_2_/Ag–SnSe_2_ system achieves an ultra-short recovery time of 1.8 × 10^−11^ s, demonstrating excellent desorption capability. These results imply that applying moderate tensile strain and operating at elevated temperatures can significantly enhance the sensor performance for SO_2_ detection.

#### 3.4.4. Recovery Time of H_2_S/Ag–SnSe_2_ and H_2_S/Au–SnSe_2_ Under Strain from −8% to 6% at Different Temperatures

**Figure 14:**
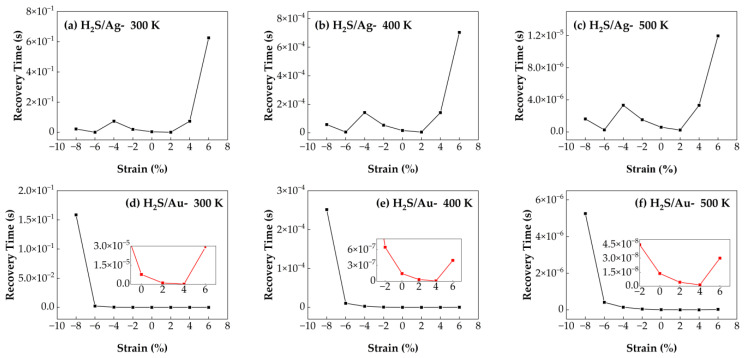
Recovery times of H_2_S adsorption on Ag- and Au-doped SnSe_2_ monolayers under biaxial strains ranging from −8% to 6% at different temperatures: (**a**–**c**) Ag-doped SnSe_2_ at (**a**) 300 K, (**b**) 400 K, and (**c**) 500 K; (**d**–**f**) Au-doped SnSe_2_ at (**d**) 300 K, (**e**) 400 K, and (**f**) 500 K.

The effect of biaxial strain (ranging from −8% to 6%) on the recovery time of H_2_S molecules adsorbed on Ag- and Au-doped SnSe_2_ monolayers is illustrated in Figure 14. For the H_2_S/Au–SnSe_2_ system, the maximum recovery time occurs under −8% compressive strain, indicating that excessive strain inhibits gas desorption and is unfavorable for sensor performance. Under tensile strain, particularly at 4%, the system exhibits optimal recovery behavior, suggesting that moderate tensile strain facilitates desorption and improves sensing performance. In contrast, the recovery time of the H_2_S/Ag–SnSe_2_ system exhibits more pronounced fluctuations under varying strain conditions. Strains of −6% and 2% result in shorter recovery times compared to the unstrained configuration. Notably, at 500 K and 2% tensile strain, the Ag–SnSe_2_ system achieves the shortest recovery time for H_2_S desorption. These results indicate that applying mild tensile strain and operating at elevated temperatures can enhance the recovery performance of H_2_S sensing on Ag-doped SnSe_2_, providing a feasible strategy for developing high-efficiency gas sensors.

#### 3.4.5. Recovery Time of HCN/Ag–SnSe_2_ and HCN/Au–SnSe_2_ Under Strain from −8% to 6% at Different Temperatures

**Figure 15:**
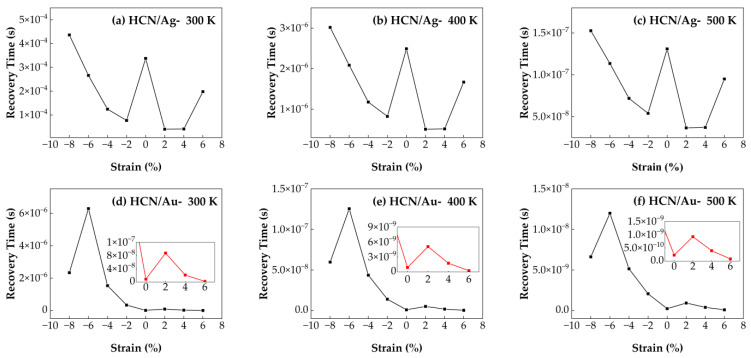
Recovery times of HCN adsorption on Ag- and Au-doped SnSe_2_ monolayers under biaxial strains ranging from −8% to 6% at different temperatures: (**a**–**c**) Ag-doped SnSe_2_ at (**a**) 300 K, (**b**) 400 K, and (**c**) 500 K; (**d**–**f**) Au-doped SnSe_2_ at (**d**) 300 K, (**e**) 400 K, and (**f**) 500 K.

The influence of biaxial strain in the range of −8% to 6% on the recovery time of HCN molecules adsorbed on Au- and Ag-doped SnSe_2_ monolayers was analyzed by calculating the desorption times at three different operating temperatures. As shown in Figure 15, the recovery time for both systems decreases with increasing temperature, indicating that thermal activation facilitates gas desorption. For the HCN/Au–SnSe_2_ system, the shortest recovery time is observed under 6% tensile strain, suggesting that tensile deformation significantly enhances desorption efficiency. In the case of HCN/Ag–SnSe_2_, 2% tensile strain leads to the best recovery performance. These results indicate that applying appropriate tensile strain can effectively regulate the desorption dynamics of HCN molecules, thereby improving sensor responsiveness. Moreover, the HCN/Ag–SnSe_2_ system shows improved recovery times under both moderate compressive strain (−6% to −2%) and tensile strain (2% to 6%) compared to the unstrained case. Conversely, for the HCN/Au–SnSe_2_ system, compressive strain adversely affects desorption, leading to longer recovery times. Overall, strain engineering offers a viable strategy for tuning gas-sensing behavior, especially for HCN molecules.

## 4. Discussion

In this study, the gas sensing properties of noble metal (Au and Ag) doped SnSe_2_ monolayers were systematically investigated using first-principles calculations. The adsorption configurations, adsorption energies, electronic properties, charge transfer, and differential charge density were evaluated for five gas molecules (NO, NO_2_, HCN, SO_2_, and H_2_S). For Au-doped SnSe_2_, the magnitude of the adsorption energies decreases in the order of NO_2_ > H_2_S > NO > HCN > SO_2_, whereas for Ag-doped SnSe_2_, the sequence is NO_2_ > H_2_S > HCN > NO > SO_2_. Among all gases, NO_2_ adsorption induces the highest charge transfer on both doped systems, indicating their promising selectivity and sensitivity toward NO_2_ molecules. In contrast, lower charge transfer values are observed for H_2_S and HCN on Au-doped SnSe_2_ and for SO_2_ and HCN on Ag-doped SnSe_2_. The differential charge density plots further visualize the electron gain or depletion at the interface, which agrees with Bader charge analysis results.

Furthermore, the effects of biaxial strain (ranging from −8% to 6%) on adsorption behavior were explored by evaluating changes in equilibrium height, adsorption energy, charge transfer, and recovery time for ten representative adsorption systems. The results reveal that both compressive and tensile strains can enhance the interaction strength between the gas molecules and the doped SnSe_2_ monolayers. Specifically, H_2_S/Au–SnSe_2_ and HCN/Au–SnSe_2_ are highly responsive to tensile strain, while NO/Au–SnSe_2_, H_2_S/Ag–SnSe_2_, NO/Ag–SnSe_2_, and NO_2_/Ag–SnSe_2_ are more sensitive to compressive strain. Systems such as NO_2_/Au–SnSe_2_, SO_2_/Au–SnSe_2_, and SO_2_/Ag–SnSe_2_ show a response to both types of strain, while HCN/Ag–SnSe_2_ is relatively insensitive in terms of charge transfer. Recovery time analysis indicates that NO_2_ has the slowest desorption kinetics among the studied gases and is most affected by strain modulation. However, increasing the operating temperature and applying appropriate strain can significantly reduce recovery time. While other gas systems exhibit less dramatic variations, strain engineering remains an effective strategy to fine-tune desorption characteristics and improve overall sensor performance. Overall, these findings provide theoretical guidance for the development of high-performance, strain-tunable gas sensors based on two-dimensional noble metal-doped SnSe_2_ monolayers.

## 5. Conclusions

In this work, we systematically investigated the gas sensing properties of Au- and Ag-doped SnSe_2_ monolayers toward five representative hazardous gases (NO, NO_2_, HCN, SO_2_, and H_2_S) using first-principles calculations. Adsorption configurations, adsorption energies, charge transfer, electronic properties, and differential charge density analyses revealed that NO_2_ exhibits the strongest interaction and highest charge transfer in both doped systems, indicating excellent selectivity and sensitivity. Biaxial strain was further applied to modulate adsorption behavior, demonstrating that both compressive and tensile strains can enhance gas–substrate interactions. Specific gas–substrate systems show varying sensitivity to strain: H_2_S/Au–SnSe_2_ and HCN/Au–SnSe_2_ respond strongly to tensile strain, while NO/Au–SnSe_2_, H_2_S/Ag–SnSe_2_, NO/Ag–SnSe_2_, and NO_2_/Ag–SnSe_2_ are more responsive to compressive strain. Recovery time analysis indicates that NO_2_ has the slowest desorption kinetics, while other gases exhibit faster recovery, and the recovery can be further optimized by adjusting operating temperature or applied strain. Overall, these findings provide a systematic framework for understanding and predicting the gas sensing performance of noble metal-doped SnSe_2_ monolayers and offer theoretical guidance for designing high-performance, strain-tunable 2D gas sensors for environmental monitoring and health protection.

## Figures and Tables

**Table 1:** Comparative summary of first-principles studies on modified SnSe_2_ systems for gas sensing applications, including target gases, modification strategies, adsorption energies, charge transfer, and recovery characteristics.

Author(s)	Year	Target Gas (es)	Modification Strategy	Adsorption Energy (eV)	Charge Transfer (e)	Recovery Time (s)	Ref.
Yuan T.	2025	CO	Pd-SnSe_2_	−0.816	0.32	65.51 (298 K)	[5]
			Ag-SnSe_2_	−0.373	0.3	2.02 × 10^−6^ (298 K)	
			Pt-SnSe_2_	−1.012	0.25	1.28 × 10^5^ (298 K)	
			Au-SnSe_2_	−0.279	0.32	5.21 × 10^−8^ (298 K)	
Xu B.	2024	CO	S-SnSe_2_	−0.1207	−0.005	1.066 × 10^−10^	[6]
		H_2_S		−0.1861	−0.046	1.335 × 10^−9^	
		NH_3_		−0.1783	−0.076	9.889 × 10^−10^	
		NO_2_		−0.2494	0.061	1.540 × 10^−8^	
		SO_2_		−0.1739	0.011	8.328 × 10^−10^	
Hung C.	2023	H_2_	SnSe	−0.085	0.017	–	[7]
		N_2_		−0.138	0.036	–	
		CO		−0.171	0.061	–	
		CO_2_		−0.201	0.041	–	
		H_2_S		−0.301	0.039	–	
		H_2_O		−0.312	0.035	–	
		NH_3_		−0.357	0.031	–	
		NO		−0.444	0.994	–	
		SO_2_		−0.596	0.390	–	
		NO_2_		−1.430	1.124	–	
Lin L.	2022	CO	Au-SnSe_2_	−0.440	−0.012		[8]
		H_2_O		−0.464	−0.044		
		NO		−0.552	0.116		
		CO	Au-SnSe_2_ under −8% biaxial strain	−0.803	−0.023	4	
		H_2_O		−0.526	−0.070	<1	
		NO		−0.823	0.202	7	
Cheng W.	2020	NO_2_	Se-Vacancy	−1.84	−0.926	–	[9]
			O-SnSe_2_	−0.32	−0.145	–	
			N-SnSe_2_	−2.98	−0.368	–	
		NH_3_	Se-Vacancy	−0.82	0.016	–	
			O-SnSe_2_	−0.13	0.000	–	
			N-SnSe_2_	−0.36	0.215	–	

**Table 2:** Optimized structural parameters of Ag- and Au-doped SnSe_2_ monolayers upon adsorption of five gas molecules.

Model		$E_{ad}$ (eV)	Height (Å)	Bond Length (Å)	Bond Angle (°)	ΔQ(e)
Ag-doped	NO	−0.29	2.104	d_N-O_ = 1.17614	---	0.140
	NO_2_	−1.03	2.185	d_NO1_ = 1.23768 d_NO2_ = 1.24053	124.9	0.540
	SO_2_	−0.13	2.649	d_SO1_ = 1.45459 d_SO2_ = 1.45398	119.5	0.050
	H_2_S	−0.57	2.612	d_SH1_ = 1.35217 d_SH2_ = 1.35257	92.1	−0.160
	HCN	−0.51	2.243	d_C-N_ = 1.15768 d_C-H_ = 1.07594	179.3	−0.004
Au-doped	NO	−0.37	2.012	d_N-O_ = 1.179	---	0.171
	NO_2_	−1.12	2.104	d_NO1_ = 1.264 d_NO2_ = 1.264	114.9	0.654
	SO_2_	−0.15	2.657	d_SO1_ = 1.454 d_SO2_ = 1.454	118.7	0.225
	H_2_S	−0.41	2.659	d_SH1_ = 1.352 d_SH2_ = 1.352	92.1	−0.041
	HCN	−0.23	2.405	d_C-N_ = 1.075 d_C-H_ = 1.159	179.8	−0.043

## Data Availability

The raw/processed data required to reproduce these findings cannot be shared at this time as the data also forms part of an ongoing study.
